# Endoscopic Cyclophotocoagulation as a Minimally Invasive Glaucoma Surgery in Clinical Practice: Appropriate for All Stages?

**DOI:** 10.7759/cureus.87693

**Published:** 2025-07-10

**Authors:** Masaki Tanito

**Affiliations:** 1 Department of Ophthalmology, Shimane University Faculty of Medicine, Izumo, JPN

**Keywords:** age-related decline, aqueous humor suppression, ciliary body, endoscopic cyclophotocoagulation, intraocular pressure, minimally invasive glaucoma surgery

## Abstract

Endoscopic cyclophotocoagulation (ECP), originally developed for the treatment of refractory glaucoma, has seen expanding use in recent years as a minimally invasive glaucoma surgery (MIGS), particularly when combined with cataract extraction. ECP reduces intraocular pressure (IOP) by suppressing aqueous humor production through targeted ablation of the ciliary processes under endoscopic guidance. This contrasts fundamentally with outflow-enhancing MIGS, which aim to restore impaired physiological function. Theoretical modeling suggests that reductions in aqueous production following ECP may approximate changes that occur over several decades of natural aging. While ECP spares the conjunctiva and is repeatable, its irreversible suppression of aqueous production may raise long-term physiological concerns. Given that aqueous humor supports the metabolic environment of anterior segment structures, sustained suppression may impact ocular homeostasis. Although clinically noteworthy complications remain rare, the growing use of ECP in non-refractory cases necessitates careful consideration. Especially as green-laser ECP is newly introduced in Japan and primarily used for refractory glaucoma, I believe it is important to revisit the fundamental differences between outflow- and inflow-targeting procedures and evaluate the broader implications of routine ECP application across glaucoma stages.

## Editorial

Endoscopic cyclophotocoagulation (ECP) has emerged as a widely adopted minimally invasive glaucoma surgery (MIGS) technique, particularly in combination with cataract extraction for patients with early to moderate primary open-angle glaucoma (POAG). Initially approved in the United States in 1991 for refractory glaucoma, ECP is now used beyond its original indication. Real-world data from the IRIS^®^ Registry show that 94% of ECP procedures are performed alongside phacoemulsification with an overall complication rate of only 1% [1]. The ongoing CONCEPT trial represents the first randomized controlled trial comparing phacoemulsification plus ECP with phacoemulsification alone in patients with POAG, aiming to clarify ECP's efficacy as a MIGS option for non-refractory glaucoma [2]. Cost-effectiveness analyses have also shown that ECP offers favorable long-term economic advantages, particularly in healthcare systems such as the NHS, due to the reusability of probes and avoidance of expensive implants [3]. Unlike filtering surgeries, ECP lowers intraocular pressure (IOP) by reducing aqueous humor production through direct visualization and ablation of the ciliary processes. Because ECP spares the conjunctiva, it can be safely combined with other angle-based MIGS procedures, such as trabecular microbypass stents or goniotomy/trabeculotomy, and does not preclude future trabeculectomy or tube shunt surgery if needed. ECP is also well-suited for cases where filtering surgery is contraindicated, such as in eyes with scleral thinning [4,5].

Despite these favorable features, it remains important to consider the physiological consequences of ECP. By thermally ablating the ciliary processes, ECP effectively reduces aqueous humor production (ΔF). Using the Goldmann equation [6], IOP = (F - U)/C + Pv, and assuming that outflow facility (C), uveoscleral outflow (U), and episcleral venous pressure (Pv) remain constant pre- and postoperatively, the change in aqueous production can be estimated by ΔF = C × (IOP_pre - IOP_post), where IOP_pre refers to the IOP before the intervention, and IOP_post refers to the IOP after the intervention. Applying this to data from published clinical studies, Francis et al. reported a typical IOP reduction of 3 mmHg following phacoemulsification combined with ECP. Assuming a standard outflow facility (C) of 0.2 µL/min/mmHg, this corresponds to ΔF of 0.6 µL/min (ΔF = 0.2 × 3) [7]. Kahook et al. observed a larger IOP reduction of 11 mmHg after ECP, which translates to a ΔF of 2.2 µL/min using the same C value (ΔF = 0.2 × 11) [8]. Of course, these estimates should be interpreted with caution, as they are based on simplified assumptions and do not take into account important factors such as baseline IOP levels, severity of glaucoma, changes in medications, variations in outflow facility, or extent of postoperative inflammation.

**Figure 1:**
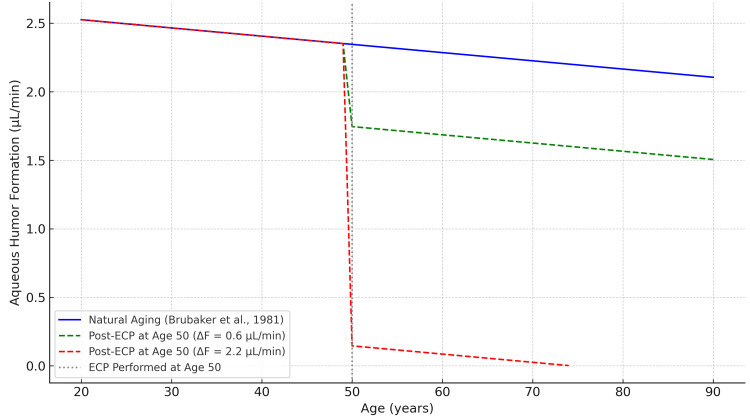
Simulated trajectories of aqueous humor formation with age, based on the Brubaker et al. model (F = -0.006 × age + 2.646) The blue line represents natural age-related decline [9]. Green and red dashed lines represent scenarios in which ECP is performed at age 50, leading to a permanent reduction of aqueous production by 0.6 and 2.2 µL/min, respectively. ECP is assumed to produce an immediate, irreversible drop in production. Curves are truncated when formation falls to zero. This figure was generated using ChatGPT-4o (OpenAI) and has not been previously published. ECP: endoscopic cyclophotocoagulation

Brubaker et al. analyzed aqueous humor formation in healthy individuals aged 20 to 83 years and proposed a linear relationship between age and aqueous production, with an average rate of decline of 0.006 µL/min/year. Based on the mean age (41 years) and mean aqueous flow rate (2.4 µL/min) described in their study, a linear regression model was estimated as F_age = -0.006 × age + 2.646, where F_age represents the estimated aqueous humor flow rate (in µL/min) at a given age [9]. The number of years of aging equivalent to a given reduction in aqueous humor production can be calculated by dividing the reduction in flow (ΔF) by the age-related decline rate of 0.006 µL/min/year. For instance, a reduction of 0.6 µL/min corresponds to 100 years of aging. This model allows for contextualizing the physiological impact of ECP-induced suppression in terms of accelerated aging (Figure 1). This irreversible suppression of aqueous formation contrasts with the mechanism of outflow-based MIGS procedures. Once aqueous production is reduced by ECP, it cannot be restored, and this may pose long-term concerns, particularly in younger patients or those with otherwise stable IOP control.

Aqueous humor is not merely responsible for maintaining IOP; it also provides essential metabolic support to tissues such as the lens, iris, and corneal endothelium [10]. A sustained reduction in aqueous volume and turnover could, in theory, compromise ocular homeostasis. While this concern is particularly relevant to procedures that suppress aqueous production, it is worth noting that surgeries designed to enhance outflow may also affect homeostatic balance, depending on the degree and mechanism of intervention. Although clinically notable complications from ECP are uncommon [1], its widespread use prompts consideration of whether it is appropriate as a first-line MIGS approach.

ECP’s role as a repeatable, conjunctiva-sparing, and cost-efficient procedure is valuable, especially in cases where filtration surgery is not feasible [4,5]. However, the growing practice of using ECP as a routine adjunct in mild or moderate glaucoma calls for careful consideration. In glaucoma, elevated IOP is often caused by a reduction in aqueous humor outflow. Surgical procedures that lower IOP by enhancing outflow aim to restore the impaired physiological function. In contrast, procedures that reduce aqueous humor production achieve IOP reduction by suppressing an existing function. In this regard, outflow-enhancing surgeries and aqueous-suppressing surgeries are fundamentally different in nature. If ECP is to be performed not only for refractory glaucoma but also for cases in which alternative treatment options exist, its use may require thoughtful justification. Comprehensive studies that assess the lifelong impact on ocular surface health, corneal endothelial cell density, and metabolic consequences of reduced aqueous production remain necessary.

In Japan, ECP using green laser was just introduced in 2022, with current indications primarily focused on refractory glaucoma-mirroring its initial clinical use in the United States [11]. While such discussions may have already taken place in regions where ECP has been used more extensively, I felt it was important to share these physiological concerns and conceptual inconsistencies. As a glaucoma specialist and developer of green laser ECP, still accumulating clinical experience with this technology, I believe this perspective may offer value to the ongoing dialogue surrounding its appropriate use. In particular, a decline in aqueous humor production is itself a hallmark of aging, and I believe it is important to recognize that ECP is a procedure that artificially induces this age-related change in the eye.
